# Evaluation of Hand Bioburden and Genomic Features of *Staphylococcus* spp. Following Handwashing Practice Among Aboriginal Schoolchildren in Rural Malaysia

**DOI:** 10.3390/tropicalmed11100266

**Published:** 2026-09-25

**Authors:** Ling Huey Khong, Nik Mohd Noor Nik Zuraina, Choo Yee Yu, Zeti Norfidiyati Salmuna, Muhammad Nashrul Farhan Samsudin, Siti Rohajar Mat Yusof, Ayub Mohamad, Norlizaini Mohd Dom, Alyaa Farhan Zulkefli, Wil Sern Lim, Terrence Anak Kuta, Muhammad Haris Fadillah Muhamad, Nur Syafawati Ismail, Yasmin Khairani Muhammad Ismadi, Tuan Noorkorina Tuan Kub

**Affiliations:** 1 Department of Medical Microbiology and Parasitology, School of Medical Sciences, Health Campus, Universiti Sains Malaysia, Kubang Kerian 16150, Malaysia; khonglinghuey@student.usm.my (L.H.K.); zetifidiyati@usm.my (Z.N.S.); wilsern91@gmail.com (W.S.L.);; 2 Medical Microbiology and Parasitology Laboratory, Hospital Pakar Universiti Sains Malaysia, Health Campus, Universiti Sains Malaysia, Kubang Kerian 16150, Malaysia; 3 Laboratory of Vaccine and Biomolecules, Institute of Bioscience, Universiti Putra Malaysia, Serdang 43400, Malaysia; 4 Bacteriology Unit, Infectious Disease, Research Centre for Molecular Research (IMR), National Institute of Health, Shah Alam 40170, Malaysia

**Keywords:** hand hygiene, Aboriginal schoolchildren, *Staphylococcus arlettae*, antimicrobial resistance, whole-genome sequencing, hand contamination, culturable bacterial bioburden

## Abstract

Data integrating hand bioburden and antimicrobial resistance with genomic characterisation among Aboriginal schoolchildren remains limited. Hand imprint samples were collected from 60 Orang Asli schoolchildren in Kelantan, Malaysia, immediately before and after handwashing, three months after a one-day educational intervention. Selected pre-handwashing *Staphylococcus* isolates underwent antimicrobial susceptibility testing, and four multidrug-resistant *Staphylococcus arlettae* isolates underwent whole-genome sequencing. Mean left-hand and median right-hand bioburden, expressed as log_10_ colony forming units(CFUs)/plate, were lower after handwashing, but neither comparison was statistically significant after Holm adjustment. *Escherichia coli*/*Shigella* complex recovery decreased from 21/60 participants (35.0%) to none (Holm-adjusted *p* < 0.001). Resistance to penicillin, fusidic acid and erythromycin was frequent, while most isolates were susceptible to linezolid and rifampicin. Sequencing identified *msr(A)* and *dfrG* in all four isolates, *mph(C)* in three, partial arginine catabolic mobile element (ACME)-associated sequences in all four, and the rep24c replicon belonging to the replication initiation protein family RepA_N in two. Genotype–phenotype concordance was partial: ResFinder detected no corresponding acquired resistance determinants for several resistant or intermediate phenotypes. These exploratory findings describe immediate bacterial recovery and selected isolates’ resistance and genomic features; they do not establish the effectiveness of the earlier educational intervention.

## 1. Introduction

Hand hygiene is one of the most effective and cost-efficient measures for preventing the transmission of infectious diseases. Handwashing with soap and water removes dirt, transient microorganisms, and reduces the burden of resident skin flora, therefore interrupting the spread of pathogens within communities and healthcare settings [1]. Despite its simplicity, inadequate hand hygiene remains a major public health concern in low- and middle-income countries, where infectious diseases continue to contribute substantially to childhood morbidity and mortality [2,3].

Children are especially vulnerable to hand contamination because of frequent contact with soil, shared objects, and environmental surfaces during daily activities [4]. Among Aboriginal populations in Malaysia, this risk may be further increased by limited access to clean water, inadequate sanitation facilities, and insufficient hygiene education [5]. Consequently, school-based hand hygiene interventions are recognised as practical public health strategies because schools provide an environment in which healthy behaviours can be reinforced through routine practice, supervision, and peer influence [6]. Previous studies have demonstrated that handwashing interventions reduce the incidence of diarrhoeal and respiratory infections among schoolchildren [7,8].

Culturable bacterial bioburden describes the quantity of bacteria recovered under specified culture conditions, while species identification describes the composition of the recovered organisms. Neither measurement alone establishes persistent bacterial carriage or colonisation. Studies have identified potentially pathogenic bacteria on children’s hands, including *Staphylococcus aureus*, coagulase-negative *Staphylococcus* spp. (CoNS), *Escherichia coli*, *Klebsiella* spp., and *Enterococcus* spp. [9,10]. Increasing evidence also indicates that healthy schoolchildren may carry antimicrobial-resistant bacteria, highlighting the potential role of hands in community dissemination of antimicrobial resistance (AMR) and the importance of antimicrobial susceptibility surveillance in community settings [11,12]. These bacteria may also possess biofilm-forming ability, virulence-associated determinants, and adaptive genomic features that contribute to persistence and transmission. Characterising selected phenotypic and genomic features can provide additional information on antimicrobial-resistant bacteria recovered from children’s hands.

Among hand-associated bacteria, *Staphylococcus* spp. are of particular interest because of their ability to colonise human skin and acquire antimicrobial resistance and virulence-associated traits [13]. Alongside *S. aureus*, coagulase-negative staphylococci (CoNS) are recognised as opportunistic pathogens, particularly in device-associated and bloodstream infections and among immunocompromised individuals [14]. Although commonly regarded as commensals, CoNS can harbour clinically important resistance determinants and serve as reservoirs for their transfer among staphylococci [14,15]. Biofilm formation can support bacterial attachment and persistence, whereas mobile genetic elements, including plasmids, can facilitate the acquisition and transfer of resistance and virulence-associated genes. These characteristics provide a rationale for investigating selected phenotypic and genomic features of hand-associated staphylococci. Molecular characterisation not only can complement phenotypic testing by identifying antimicrobial resistance determinants, other genomic features of selected *Staphylococcus* isolates can also be detected.

Although previous studies have demonstrated the effectiveness of hand hygiene interventions in reducing microbial contamination, relatively few have combined immediate pre- and post-handwashing microbiological assessments with detailed characterisation of antimicrobial-resistant hand-associated bacteria, particularly among Aboriginal children. Conventional phenotypic antimicrobial susceptibility testing (AST) provides limited information on the genetic characteristics of resistant isolates. Whole-genome sequencing (WGS) can complement phenotypic testing by identifying antimicrobial resistance determinants and other genomic features potentially associated with bacterial adaptation and pathogenic potential.

Previous genomic studies of *Staphylococcus* spp. have reported antimicrobial resistance genes such as *msr(A)*, *dfrG* and *mph(C)* [16,17], as well as arginine catabolic mobile element (ACME)-associated sequences, plasmid-associated replication features such as the replication initiation protein family RepA_N, and biofilm-associated determinants [18,19,20]. These findings provide a basis for investigating selected genomic features of less-characterised staphylococci recovered from community settings. In the present study, genomic analyses focused on acquired antimicrobial resistance genes, virulence-associated sequence matches, plasmid replicons and predicted human pathogenic potential. Biofilm- and adhesion-associated genes were not systematically analysed.

The paired hand-imprint cultures and isolate characterisation provide complementary information from the same microbiological assessment. The paired cultures describe immediate changes in culturable bacterial recovery following handwashing, while antimicrobial susceptibility testing and sequencing characterise selected bacteria recovered before washing. Together, these analyses describe hand-associated bacteria at follow-up but do not evaluate longitudinal changes in antimicrobial resistance or genomic characteristics attributable to education.

Therefore, this study aimed to evaluate immediate changes in culturable hand bacterial bioburden and the frequency of bacterial detection following handwashing performed three months after a one-day hand hygiene educational intervention among Aboriginal (Orang Asli) schoolchildren in Gua Musang, Kelantan, Malaysia. Additional objectives were to determine the antimicrobial susceptibility profiles of selected pre-handwashing *Staphylococcus* isolates and to characterise acquired antimicrobial resistance genes, virulence-associated sequence matches, plasmid replicons and predicted human pathogenic potential in four selected multidrug-resistant *Staphylococcus arlettae* isolates using WGS.

## 2. Materials and Methods

### 2.1. Study Design

This uncontrolled, single-arm study included a paired microbiological assessment immediately before and after handwashing during a follow-up visit three months after a hand hygiene educational session. The project was conducted from August to October 2025. As this is a community service program, no microbiological samples were collected before the educational session, and no control group was included.

### 2.2. Study Population and Setting

The study was conducted among 60 Aboriginal (Orang Asli) primary school students (Year 1–Year 6; 7–12 years old) at Sekolah Kebangsaan Pasir Linggi, Gua Musang, Kelantan, Malaysia.

The school was selected because it serves Aboriginal children from surrounding villages as well as more distant communities within the Gua Musang district, including Kampung Kuala Koh and Kampung Aring, which were located approximately 100 km from the school. Students from distant villages resided in the school hostel during the academic term.

Administrative permission to conduct the study was obtained from the Kelantan State Education Department and the school principal. These permissions were separate from parental or guardian consent and individual child assent.

Of 63 pupils approached, written parental consent was obtained for all 63. Two pupils were excluded because they could not understand Malay sufficiently to meet the study eligibility criteria. The remaining 61 pupils provided verbal assent and were enrolled. Each child’s verbal assent was documented by the facilitator in the study records. One pupil was absent at the three-month follow-up visit, leaving 60 participants with paired microbiological samples for analysis, comprising 27 hostel pupils and 33 day pupils. Participant flow is presented in Figure 1.

### 2.3. Inclusion and Exclusion Criteria

Students from Year 1 to Year 6 whose parents or legal guardians provided written informed consent were eligible for inclusion. Students who were absent during the intervention or follow-up sessions or were unable to communicate in Malay were excluded.

Verbal assent was sought separately from each child by a facilitator using standardised guidance. The facilitator explained the study procedures individually in language the child could understand, asked whether the child agreed to participate and recorded the child’s response accordingly.

### 2.4. Sample Size

Sample-size calculation was performed using G*Power version 3.1.9.4. Because sufficient estimates of means and standard deviations (SDs) were unavailable from previous studies, an assumed standardised paired effect size of d_z_ = 0.40 was used. An a priori calculation for a two-sided paired *t*-test, with a significance level of 0.05 and 80% power, required 52 participants with complete paired measurements. Allowing for 10% dropout corresponds to a minimum recruitment requirement of 58 participants (52/0.90, rounded up). The present microbiological analysis included 60 participants with paired samples. The original sample-size planning did not designate a single primary endpoint and did not establish power for the individual Wilcoxon signed-rank or McNemar comparisons or for multiplicity-adjusted analyses. The microbiological comparisons are therefore presented as exploratory analyses.

### 2.5. Hand Hygiene Intervention and Sample Collection

The hand hygiene intervention was delivered on a single day through one educational session lasting approximately one hour, comprising 55 min of scheduled educational and practical activities. The session had two components: a 30 min PowerPoint presentation explaining the importance of hand hygiene and handwashing steps based on World Health Organization (WHO) [21] and Centers for Disease Control and Prevention (CDC) [22] guidelines, followed by practical training comprising a 15 min demonstration and 10 min of supervised handwashing practice. The educational objectives were to explain the role of hand hygiene in preventing infections, increase awareness of correct handwashing, and teach techniques for use in daily routines.

Baseline hand hygiene knowledge was not assessed in this study. A hand hygiene talk had previously been delivered by the Ministry of Health Malaysia in 2020 during the coronavirus disease 2019 (COVID-19) pandemic. However, participants’ attendance at that activity and retention of its content were not assessed; therefore, their previous exposure to hand hygiene education and baseline knowledge could not be established.

One main presenter from the Department of Medical Microbiology and Parasitology delivered the educational content, and 10 facilitators guided assigned groups. All facilitators were trained in the correct handwashing steps before teaching and supervising the children. The educational session was delivered in Malay language to support understanding among the Orang Asli children. Practical training was conducted at designated stations using water tanks and soap supplied by the research team. The 10 min practical component represented the time allocated to the activity rather than the duration of each participant’s handwashing.

To support continued practice, the research team supplied the school with 100 bottles of soap, each containing 100 mL. Two bottles were placed at each designated handwashing location, including the canteen and toilet sinks. During the three-month interval, teachers were asked to periodically observe students’ handwashing and provide small gifts supplied by the research team when students performed the technique correctly. This arrangement was adopted because the school’s remote location and travel difficulties limited frequent visits by the research team. Soap and water availability were not systematically monitored throughout this interval; however, at the three-month follow-up visit, soap and clean running water were available. To maintain a streamlined study design, formal quantitative metrics for intervention fidelity and routine handwashing behaviour were outside the current scope. Therefore, teacher-supported observation and encouragement were not collected as structured behavioural outcome data. No formal quantitative assessment of intervention fidelity or change in everyday handwashing behaviour was undertaken. Accordingly, the microbiological assessment concerned immediate differences before and after handwashing at follow-up rather than the sustained effectiveness of the educational intervention.

Three months after the training, the same participants attended a follow-up session and were identified using their study identification numbers. Hand imprint samples were collected immediately before and after independent handwashing. Before handwashing, the palm and palmar surfaces of all five digits, including the thumb, of each hand were pressed onto separate 150 mm blood agar (BA) plates (OXOID, Basingstoke, UK) for 3 s. Participants were then asked to wash both hands together using soap and water, independently applying the technique they recalled from the educational intervention. Soap use was monitored, and clean running water was available. Although participants had been taught a handwashing duration of approximately 40–60 s during the educational session, actual duration at follow-up was not monitored or recorded. The follow-up assessed microbiological outcomes surrounding a requested handwashing event rather than directly measuring routine daily handwashing behaviour. After handwashing, participants dried their hands using paper towels before repeating the imprint procedure on new BA plates, with the same hand surfaces contacting the agar for 3 s per hand. All culture plates were labelled and transported to the laboratory on the same day for microbiological processing.

### 2.6. Bacterial Analysis

After sample collection, blood agar plates were incubated aerobically at 37 °C for 18–24 h before microbiological analysis.

### 2.7. Colony Enumeration

Bacterial growth was quantified by manually counting CFUs on each hand imprint plate. All hand imprint plates were individually countable. No plates were classified as too numerous to count (TNTC), and no upper-limit values were substituted for the observed colony counts. These counts represented bacteria transferred from the sampled hand surfaces and recoverable under the specified incubation conditions. Raw counts were recorded as CFUs/plate and transformed using the base-10 logarithm before statistical analysis. Culturable bacterial bioburden was reported as log_10_ CFUs/plate, with left- and right-hand samples evaluated separately.

For species-specific analyses, detection was recorded when an identified organism was recovered from a participant’s hand imprint cultures. Hand contamination refers here to the presence of recoverable surface-associated microorganisms; the method did not distinguish resident microbiota from transient contaminants or establish persistent carriage or colonisation.

Yeast and mould growth observed incidentally on the blood agar hand imprint plates was recorded separately for each hand as detected or not detected. When yeast growth was observed, a wet mount preparation was performed to support its recognition. The culture conditions—blood agar incubated aerobically at 37 °C for 18–24 h—were intended for bacterial recovery and were not optimised for comprehensive fungal detection. These observations were therefore treated as incidental culture findings and analysed as exploratory outcomes. Non-detection under these conditions does not establish the absence of fungi.

### 2.8. Bacterial Identification

Microbiological identification focused on *Gram-positive cocci* and *Gram-negative bacilli* commonly associated with skin, gastrointestinal, and respiratory microorganisms.

Colony selection was guided by morphology and the bacterial groups targeted for investigation, without a fixed number of colonies selected per hand imprint plate. Colonies displaying appearances consistent with the target groups were picked for further isolation and identification. White, pale-yellow, golden-yellow or mucoid colonies suspected of representing *Gram-positive cocci* were subcultured onto Mannitol Salt Agar (MSA) (OXOID, Basingstoke, UK), whereas greyish or mucoid colonies suspected of representing *Gram-negative bacilli* were subcultured onto MacConkey Agar (MAC) (OXOID, Basingstoke, UK). Colony appearance was used for preliminary selection and was not considered sufficient for species identification.

Pure isolates obtained from MSA and MAC were identified using matrix-assisted laser desorption ionisation time-of-flight mass spectrometry (MALDI-TOF MS; Bruker MALDI Biotyper^®^ System, Bruker Daltonics, Bremen, Germany) according to the manufacturer’s instructions. MALDI-TOF MS identification scores ≥ 2.0 were accepted for species-level identification, whereas scores ≥ 1.7 but <2.0 were accepted for genus-level identification only. Scores < 1.7 were considered unreliable. Low-score isolates were retested, and retested unresolved identifications were excluded from analyses requiring an accepted identification.

Colonies suspected of representing enteric bacteria, including *Escherichia coli*, *Salmonella* spp., and *Shigella* spp., were additionally investigated using Deoxycholate Citrate Agar (DCA) (OXOID, Basingstoke, UK) followed by biochemical characterisation using Triple Sugar Iron (TSI) (OXOID, Basingstoke, UK), urea (OXOID, Basingstoke, UK), and Sulphide–Indole–Motility (SIM) (OXOID, Basingstoke, UK) tests. Biochemical reactions were interpreted according to the manufacturer’s instructions and standard identification criteria described in the Manual of Clinical Microbiology, 11th edition (2015). The available MALDI-TOF MS and biochemical findings did not establish a definitive species-level identification for isolates initially assigned as *S. sonnei*. These isolates were therefore reported as *E. coli*/*Shigella* complex. TSI, urea and SIM results were not considered confirmatory for differentiation within this complex.

### 2.9. Antimicrobial Susceptibility Testing

Six *Staphylococcus* species identified by MALDI-TOF MS were purposively selected for antimicrobial susceptibility testing: *S. aureus*, *S. saprophyticus*, *S. haemolyticus*, *S. arlettae*, *S. pasteuri* and *S. ureilyticus*. All pre-handwashing isolates belonging to these six species were tested; no further selection was performed within each species. Species selection aimed to include clinically important and less-characterised staphylococci recovered from the hands of Aboriginal schoolchildren. They were included because of their recognised roles as opportunistic human pathogens. *S. arlettae* was selected owing to its relatively high frequency among the hand isolates, providing an opportunity to investigate the antimicrobial susceptibility profile of an understudied coagulase-negative staphylococcal species. *S. pasteuri* and *S. ureilyticus* were included because they are commonly associated with environmental reservoirs and human skin colonisation, allowing the antimicrobial susceptibility profiles of these less-studied species recovered from children’s hands to be characterised.

Species were selected purposively, and all recovered pre-handwashing isolates belonging to the six selected species underwent AST. The tested collection comprised 71 isolates: eight *S. aureus*, 25 *S. saprophyticus*, seven *S. ureilyticus*, 20 *S. arlettae*, six *S. haemolyticus* and five *S. pasteuri*. AST percentages were calculated using the number of tested isolates of each species as the denominator. In contrast, bacterial detection frequencies were calculated at the participant level. 

Susceptibility percentages were calculated using the total number of tested isolates of each species as the denominator. These percentages describe the recovered isolates of the selected species and should not be interpreted as prevalence estimates among participants or among all hand-associated staphylococci.

Antimicrobial susceptibility testing was performed using the Kirby–Bauer disk-diffusion method on Mueller–Hinton agar (Oxoid, Basingstoke, UK). The antimicrobial panel was selected with reference to the agents used for testing clinical isolates in the Medical Microbiology and Parasitology laboratory at Universiti Sains Malaysia. Prescribing practices and antimicrobial consumption in the study community were not assessed. The panel comprised penicillin G (10 U), linezolid (30 μg), trimethoprim/sulfamethoxazole (1.25/23.75 μg), fusidic acid (10 μg), novobiocin (5 μg), clindamycin (2 μg), cefoxitin (30 μg), rifampicin (5 μg) and erythromycin (15 μg). All antimicrobial disks were obtained from Bioanalyse (Ankara, Turkey).

For each isolate–antimicrobial combination, disk-diffusion testing was performed on three replicate plates. Inhibition zone diameters for penicillin, linezolid, trimethoprim/sulfamethoxazole, clindamycin, erythromycin and rifampicin were measured in millimetres, and the arithmetic mean of the three measurements was rounded before assigningthe final susceptible, intermediate or resistant category according to the Clinical and Laboratory Standards Institute (CLSI) M100, 35th edition (2025).

Novobiocin susceptibility was assessed by disk diffusion according to CLSI M02 methodology. As CLSI M100 does not provide current clinical breakpoints for novobiocin against *Staphylococcus* spp., zone diameters were interpreted using a previously published criterion of ≥16 mm for susceptible and <16 mm for resistant [23]. 

The criteria applicable to all staphylococci were applied to the six tested species. Cefoxitin was interpreted as a surrogate marker of methicillin (oxacillin) resistance using the CLSI criteria for *S. aureus* and the applicable category for other *Staphylococcus* spp. Fusidic acid was interpreted using the *Staphylococcus* disk-diffusion breakpoints in the European Committee on Antimicrobial Susceptibility Testing (EUCAST) version 15.0 (2025).

For this study, multidrug-resistant (MDR) classification was adapted from the framework of Magiorakos et al. (2012) and defined as phenotypic non-susceptibility to at least one agent in three or more eligible antimicrobial categories [24]. CLSI intermediate and resistant categories were counted as non-susceptible, while the individual categories remained separately reported. The eligible tested categories were oxazolidinones (linezolid), folate pathway inhibitors (trimethoprim/sulfamethoxazole), fucidanes (fusidic acid), lincosamides (clindamycin), antistaphylococcal β-lactams (assessed using cefoxitin as a surrogate for methicillin resistance), ansamycins (rifampicin) and macrolides (erythromycin). Known intrinsic resistance traits and missing or uninterpretable results were excluded. The adaptation to CoNS and the tested antimicrobial panel was exploratory; MDR classification did not establish the molecular mechanism or place of acquisition of resistance.

### 2.10. Genomic DNA Extraction and Whole-Genome Sequencing

20 *Staphylococcus arlettae* isolates were recovered from pre-handwashing samples, and all 20 underwent antimicrobial susceptibility testing. 13 of the 20 tested *S. arlettae* isolates (65.0%) met the criterion of non-susceptibility to at least one agent in three or more eligible antimicrobial categories. Four of the 13 MDR isolates—208R, 209R(2), 508R(2) and 603R(3)—were selected for whole-genome sequencing based on their phenotypic antimicrobial resistance profiles. *S. arlettae* was prioritised to provide genomic information on a less-characterised staphylococcal species recovered from the children’s hands. Whole-genome sequencing was performed to investigate antimicrobial resistance determinants and other genomic features of these selected isolates.

The selected four multidrug-resistant isolates were revived from cryopreserved stocks and subcultured on blood agar to obtain pure overnight cultures. Genomic DNA was extracted using the MasterPure™ Complete DNA and RNA Purification Kit (Lucigen, Middleton, WI, USA) according to the manufacturer’s instructions, with the magnetic bead-based protocol for Gram-positive bacteria. Briefly, bacterial cells were resuspended in 300 µL of MasterPure™ lysis buffer and transferred to tubes containing magnetic beads. The samples were homogenised by bead beating for two cycles of 13 s each, followed by centrifugation at 12,000 rpm for 3 min. The resulting pellet and supernatant mixture were resuspended to facilitate DNA release and subsequently transferred to clean microcentrifuge tubes for downstream purification.

Library preparation and sequencing were performed following the manufacturer’s recommended protocols. DNA concentration during library preparation was quantified using the Qubit™ 2.0 Fluorometer (Invitrogen, Life Technologies, Carlsbad, CA, USA). Final DNA concentration and purity were assessed using a NanoDrop spectrophotometer (Thermo Fisher Scientific, Waltham, MA, USA). Samples meeting the required quality criteria, including a DNA concentration of >80 ng/µL and an A260/A280 ratio of 1.8–2.0, were subjected to whole-genome sequencing using the PromethION 2 Solo (P2 Solo) platform (Oxford Nanopore Technologies plc, Oxford, UK).

### 2.11. Bioinformatics Analysis

Bioinformatics analysis was performed to characterise acquired antimicrobial resistance determinants, virulence-associated sequence matches and plasmid replicons in four selected *Staphylococcus arlettae* isolates. Oxford Nanopore sequencing reads in FASTQ format were assembled de novo using Flye v2.9.5-b1801 with the --nano-raw option. The resulting draft assemblies were indexed using bwa index, followed by read mapping with the BWA-MEM algorithm implemented in BWA v0.7.17-r1188 using default settings. The resulting alignment files were processed using SAMtools v1.19 for coverage assessment. Sequencing depth was calculated using samtools depth -a, with the -a option including positions with zero coverage. The same Flye and BWA versions were used for all four isolates.

The draft assemblies subsequently underwent two rounds of polishing using Medaka v2.0.1 with Oxford Nanopore reads only; no short-read data were used. Medaka consensus was run using the Nanopore FASTQ reads and the consensus FASTA from the preceding polishing round, together with the --bacteria option. The separately generated BWA alignment was not used as input to the Medaka command. The final polished assemblies were exported in FASTA format for downstream analyses.

Downstream analyses were performed using the Center for Genomic Epidemiology (CGE) web-based platform. Acquired antimicrobial resistance genes were screened using ResFinder v4.7.2, virulence-associated sequence matches using VirulenceFinder v3.2.1, and plasmid replicons using PlasmidFinder v3.0.3. Exploratory prediction of human pathogenic potential was performed using PathogenFinder v0.6.0. No dedicated, systematic screening of biofilm- or adhesion-associated genes was performed. The acquired resistance-gene findings were compared with the corresponding phenotypic antimicrobial susceptibility profiles to assess genotype–phenotype concordance. Database matches were interpreted according to nucleotide identity, alignment length and reference-sequence coverage. These measures were not considered sufficient, individually or together, to establish gene integrity, expression or function. Partial ACME-associated matches were not interpreted as evidence of an intact mobile element.

### 2.12. Statistical Analysis

Statistical tests were performed using IBM SPSS Statistics version 30.0. Adjustment for multiple comparisons was calculated in Microsoft Excel 2024 (Microsoft Corporation, Redmond, WA, USA).

The original study protocol did not designate a single primary endpoint or specify a primary hand-specific or combined-hand CFU measure. The microbiological comparisons in this report are therefore presented as exploratory analyses. Left- and right-hand CFU outcomes were analysed separately, and Holm adjustment was applied across the microbiological comparisons.

Paired microbiological analyses included 60 participants with samples collected immediately before and after handwashing at the three-month follow-up visit. One pupil attended the educational session but was absent at follow-up and therefore contributed no microbiological measurements. These missing measurements were not treated as zero counts or negative cultures.

Bacterial CFU counts were log_10_-transformed before analysis. Normality was assessed for the paired differences between post- and pre-handwashing log-transformed counts separately for each hand. Left-hand values were summarised as mean ± standard deviation and compared using a paired *t*-test. Right-hand values were summarised as median (first quartile [Q1]–third quartile [Q3]) and compared using the Wilcoxon signed-rank test.

Left- and right-hand outcomes were analysed separately, with pre- and post-handwashing measurements paired within each participant. Measurements from the two hands were not pooled as independent observations. No direct statistical comparison of changes between hands was performed.

Yeast and mould detection was analysed as exploratory binary outcomes, coded as detected or not detected for each hand at each sampling point. Frequencies and percentages were calculated using the number of participants as the denominator. Pre- and post-handwashing detection was compared separately for each hand using two-sided exact McNemar tests.

Gram-category and bacterial species detection were analysed at the participant level. Participants were classified as positive if at least one bacterium belonging to the respective category or species was recovered from either hand at the corresponding sampling point. Each participant was counted once per category or species per sampling point. Gram-category comparisons were analysed using two-sided exact McNemar tests. Species-specific comparisons were analysed using two-sided exact McNemar tests, including comparisons with low counts or a zero discordant cell.

All tests were two-sided. The Holm–Bonferroni correction was applied across a single family of 52 pre- and post-handwashing microbiological comparisons: 44 bacterial taxa, two overall Gram categories, four hand-specific yeast and mould comparisons, and two hand-specific CFU comparisons. Both unadjusted and Holm-adjusted *p*-values were reported, with statistical significance assessed using adjusted *p*-values < 0.05.

Antimicrobial susceptibility results were summarised as frequencies and percentages of susceptible, intermediate and resistant isolates for each antibiotic. Heatmaps were generated to visualise isolate-level antimicrobial susceptibility patterns.

### 2.13. Ethical Considerations

Ethical approval was obtained from the Human Research Ethics Committee (JEPeM), Universiti Sains Malaysia (Reference No. USM/JEPeM/KK/24030285). Written informed consent was obtained from the school authorities and the parents or legal guardians of the participants, while verbal assent was obtained from participating students. Participant confidentiality and anonymity were maintained throughout the study.

## 3. Results

### 3.1. Immediate Changes in Culturable Bacterial Bioburden Following Handwashing

Culturable bacterial bioburden immediately before and after handwashing at the three-month follow-up visit was analysed on the log_10_ scale (Table 1). For the left hand, mean bioburden decreased from 2.5668 log_10_ CFUs/plate (SD: 0.2212) before handwashing to 2.4624 log_10_ CFUs/plate (SD: 0.3138) afterwards. The mean paired difference, calculated as post- minus pre-handwashing, was −0.1044 log_10_ units. Although the unadjusted paired *t*-test yielded *p* = 0.021, the comparison was not statistically significant after Holm adjustment across the 52 microbiological comparisons (t(59) = −2.379; adjusted *p* = 1.000).

For the right hand, median bioburden decreased from 2.5508 log_10_ CFUs/plate (Q1–Q3: 2.3881–2.6964) before handwashing to 2.5125 log_10_ CFUs/plate (Q1–Q3: 2.3071–2.7092) afterwards. This comparison was not statistically significant before or after Holm adjustment (Wilcoxon signed-rank test, Z = −1.612; unadjusted *p* = 0.107; adjusted *p* = 1.000).

Individual responses varied: post-handwashing CFU counts were higher than pre-handwashing counts in 25 of 60 participants (41.7%) for the left hand and 24 of 60 participants (40.0%) for the right hand.

### 3.2. Incidental Yeast and Mould Detection Frequencies

Yeast and mould were incidentally recovered during hand imprint culture and were evaluated as exploratory outcomes. Their detection frequencies immediately before and after handwashing at the three-month follow-up visit are presented in Table 2. Yeast detection increased from nine (15.0%) to 12 (20.0%) participants on the left hand and from 11 (18.3%) to 18 (30.0%) on the right hand. Neither paired comparison was statistically significant after Holm adjustment (adjusted *p* = 1.000 for both hands).

Similarly, mould detection increased from 29 (48.3%) to 38 (63.3%) participants on the left hand and from 34 (56.7%) to 37 (61.7%) on the right hand. Neither paired comparison was statistically significant after Holm adjustment (adjusted *p* = 1.000 for both hands).

### 3.3. Gram Stain Distribution of Bacteria Before and After Handwashing

The proportions of participants with hand cultures positive for Gram-positive and Gram-negative bacteria immediately before and after handwashing at the three-month follow-up visit are presented in Table 3. Gram-positive bacteria were detected in 58 participants (96.7%) before handwashing and 50 participants (83.3%) afterwards. This difference was not statistically significant after Holm adjustment (adjusted *p* = 0.391).

Gram-negative bacteria were detected in 40 participants (66.7%) before handwashing and 27 participants (45.0%) afterwards. This difference was also not statistically significant after Holm adjustment (adjusted *p* = 0.939). Gram-positive bacteria were more frequently detected than Gram-negative bacteria at both sampling points.

### 3.4. Bacterial Distribution Before and After Handwashing

The distribution of bacteria detected immediately before and after handwashing at the three-month follow-up visit is presented in Table 4. Among Gram-positive bacteria, *Staphylococcus aureus* was detected in seven participants (11.7%) before handwashing and one participant (1.7%) afterwards. *Staphylococcus saprophyticus* was detected in 22 participants (36.7%) before handwashing and 13 participants (21.7%) afterwards. Neither comparison was statistically significant after Holm adjustment (adjusted *p* = 1.000 for *S. aureus* and 0.199 for *S. saprophyticus*). Other *Staphylococcus* spp., including *S. arlettae*, *S. ureilyticus* and *S. haemolyticus*, were detected in fewer participants after handwashing, but these differences were not statistically significant after Holm adjustment (adjusted *p* = 1.000 for each comparison).

Among Gram-negative bacteria, *E. coli*/*Shigella* complex was detected in 21 participants (35.0%) before handwashing and was not recovered afterwards. This difference was statistically significant after Holm adjustment (adjusted *p* < 0.001). *Klebsiella pneumoniae* was detected in six participants (10.0%) before handwashing and was not recovered afterwards, although this comparison was not statistically significant after Holm adjustment (adjusted *p* = 1.000).

Lower detection frequencies were also observed for *Acinetobacter baumannii*, *Enterobacter cloacae*, *Stenotrophomonas maltophilia* and *Pseudomonas aeruginosa*. None of these comparisons was statistically significant after Holm adjustment (adjusted *p* = 1.000 for each comparison).

### 3.5. Antimicrobial Susceptibility Profiles of Selected Staphylococcus spp. Isolates

The antimicrobial susceptibility profiles of 71 pre-handwashing *Staphylococcus* isolates representing six selected species are presented in Table 5. All recovered pre-handwashing isolates belonging to these species underwent testing. Percentages describe the tested isolates within each species and do not represent resistance prevalence among participants or all recovered hand-associated staphylococci. All 71 isolates were susceptible to linezolid. For rifampicin, 70 isolates were susceptible and one *S. ureilyticus* isolate was intermediate; no resistant isolates were detected.

Among *S. aureus*, 7/8 isolates (87.5%) were resistant to penicillin and 2/8 (25.0%) to fusidic acid. Two isolates (25.0%) showed intermediate susceptibility to erythromycin, while the remaining six were susceptible. All eight isolates were susceptible to cefoxitin, clindamycin and trimethoprim/sulfamethoxazole.

Among *S. saprophyticus*, resistance was observed to fusidic acid in 23/25 isolates (92.0%), penicillin in 4/25 (16.0%), erythromycin in 2/25 (8.0%) and clindamycin in 1/25 (4.0%). Intermediate susceptibility was observed to erythromycin in two isolates (8.0%) and clindamycin in one (4.0%). All isolates were susceptible to cefoxitin and trimethoprim/sulfamethoxazole. All seven *S. ureilyticus* isolates were resistant to fusidic acid, and two (28.6%) were resistant to erythromycin. All were susceptible to penicillin, cefoxitin, clindamycin and trimethoprim/sulfamethoxazole.

All 20 *S. arlettae* isolates were resistant to penicillin. Resistance was also observed to fusidic acid in 17/20 isolates (85.0%), erythromycin in 15/20 (75.0%), trimethoprim/sulfamethoxazole in 4/20 (20.0%), and clindamycin and cefoxitin in one isolate each (5.0%). Clindamycin results were predominantly intermediate, accounting for 15/20 isolates (75.0%), while four (20.0%) were susceptible.

Among *S. haemolyticus*, resistance was observed to erythromycin in 4/6 isolates (66.7%), penicillin in 2/6 (33.3%), and fusidic acid and cefoxitin in one isolate each (16.7%). Among *S. pasteuri*, 2/5 isolates (40.0%) were resistant to penicillin, while all five were susceptible to the remaining agents interpreted using CLSI or EUCAST criteria.

All tested *S. saprophyticus*, *S. arlettae* and *S. ureilyticus* isolates, together with one *S. aureus* isolate, were categorised as resistant to novobiocin. Novobiocin and penicillin G were excluded from the antimicrobial category count. Novobiocin was initially included to broadly profile the general susceptibility of the staphylococcal isolates, but the resistant results were ultimately excluded because the antibiotic is no longer used therapeutically and its predictable, intrinsic resistance in species like *S. saprophyticus* provides no clinically actionable treatment data. Testing of and reporting on penicillin G provides valuable epidemiological data for tracking baseline phenotypic trends; however, penicillin G was excluded from the MDR category count because penicillin resistance could not be interpreted consistently as acquired resistance across all tested *Staphylococcus* species.

Using the adapted MDR definition, 13/20 *S. arlettae* isolates (65.0%) met the classification criteria. Four isolates—208R, 209R(2), 508R(2) and 603R(3)—underwent whole-genome sequencing, representing 30.8% of the MDR isolates and 20.0% of all tested *S. arlettae* isolates.

Figure 2 illustrates variation in antimicrobial susceptibility among individual isolates within and between the selected species. Fusidic acid resistance was frequent among *S. saprophyticus*, *S. arlettae* and *S. ureilyticus*, while penicillin resistance was observed in all *S. arlettae* isolates and most *S. aureus* isolates. Clindamycin intermediate phenotypes were concentrated among *S. arlettae*. The four sequenced *S. arlettae* isolates shared the same susceptibility profile: resistance to penicillin, trimethoprim/sulfamethoxazole, fusidic acid and erythromycin; intermediate susceptibility to clindamycin; and susceptibility to linezolid, cefoxitin and rifampicin. Penicillin G and novobiocin results were reported separately and were excluded from the antimicrobial category count used for MDR classification. Similarity in these phenotypic profiles does not establish genetic relatedness or transmission between participants.

### 3.6. Genomic Characterisation of Multidrug-Resistant Isolates

#### 3.6.1. Acquired Antimicrobial Resistance Genes and Genotype–Phenotype Comparison

Whole-genome analysis of the four *Staphylococcus arlettae* isolates identified a limited number of acquired AMR genes (Table 6). All detected AMR genes showed 100% coverage, with nucleotide identities ranging from 85.82% to 100.00%. The macrolide resistance gene *msr(A)* and the trimethoprim resistance gene *dfrG* were detected in all four isolates, whereas *mph(C)* was identified only in isolates 208R, 209R(2), and 508R(2).

For isolate 208R, ResFinder detected *msr(A)*, *mph(C)*, and *dfrG*, corresponding to resistance determinants associated with macrolides and trimethoprim. No acquired resistance genes associated with β-lactams, aminoglycosides, tetracyclines, glycopeptides, oxazolidinones, fluoroquinolones, fusidic acid, rifampicin, or other antimicrobial classes were identified. Similarly, isolate 209R(2) harboured *msr(A)*, *mph(C)*, and *dfrG*. The nucleotide identities were 85.82% for *msr(A)*, 90.00% for *mph(C)*, and 100.00% for *dfrG*. Isolate 508R(2) also contained *msr(A)*, *mph(C)*, and *dfrG*. Two full-length *msr(A)* hits were identified, showing nucleotide identities of 86.78% and 99.05%, while *mph(C)* and *dfrG* exhibited 100.00% identity. In contrast, isolate 603R(3) carried only *msr(A)* and *dfrG*. The *msr(A)* gene showed 86.78% nucleotide identity, whereas *dfrG* showed 100.00% identity.

Lower-identity matches, including those with approximately 86% nucleotide identity to *msr(A)*, were interpreted as putative *msr(A)*-like sequences. Nucleotide identity and reference coverage were reported separately. Full reference coverage does not independently establish an intact coding sequence or biological function.

Comparison of antimicrobial susceptibility profiles with acquired resistance genes detected by ResFinder showed partial genotype–phenotype concordance (Table 7). All four sequenced *S. arlettae* isolates were resistant to penicillin and fusidic acid and showed intermediate susceptibility to clindamycin, but no corresponding acquired resistance determinants were detected by ResFinder. This discordance was a notable finding of the genomic investigation.

The macrolide resistance gene *msr(A)* and the trimethoprim resistance gene *dfrG* were detected in all four isolates, while *mph(C)* was detected in 208R, 209R(2) and 508R(2). These findings were consistent with the observed erythromycin and trimethoprim/sulfamethoxazole resistance phenotypes, although *dfrG* specifically encodes resistance to the trimethoprim component. The acquired resistance genes detected therefore accounted for only part of the observed phenotypic profile.

#### 3.6.2. Virulence-Associated Factor (VirulenceFinder)

Analysis of virulence-associated factors using VirulenceFinder identified only ACME-associated sequences among the four *Staphylococcus arlettae* isolates (Table 8). Multiple ACME-associated hits were detected across all isolates, with seven hits identified in isolates 208R, 508R(2), and 603R(3), and nine hits detected in isolate 209R(2). The detected sequences showed nucleotide identities ranging from 77.93% to 89.36% compared with the ACME reference sequences. These findings represent partial matches to ACME-associated regions rather than confirmation of a complete ACME element.

#### 3.6.3. Plasmid Replicon Analysis (PlasmidFinder)

PlasmidFinder analysis revealed that isolates 208R and 508R(2) carried an identical plasmid replicon, identified as rep24c belonging to the RepA_N family. In contrast, no plasmid replicon sequences were detected in isolates 209R(2) and 603R(3).

#### 3.6.4. Pathogen Probability Prediction (PathogenFinder2)

Genome-based pathogenicity prediction using PathogenFinder2 classified all four *Staphylococcus arlettae* isolates as human non-pathogenic (Table 9). The prediction scores ranged from 0.3259 ± 0.1261 to 0.4538 ± 0.1176, indicating a low predicted likelihood of human pathogenicity. Among the isolates, 508R(2) showed the highest prediction score (0.4538 ± 0.1176), whereas 603R(3) showed the lowest score (0.3259 ± 0.1261). Overall, no isolate was predicted to have a pathogenic phenotype based on the PathogenFinder2 classification.

## 4. Discussion

### 4.1. Immediate Changes in Culturable Hand Bacterial Bioburden Following Handwashing

This study evaluated immediate microbiological changes following handwashing performed three months after a one-day hand hygiene educational intervention. Culturable bacterial bioburden which was reported as log_10_ CFUs/plate found that mean left-hand and median right-hand values were lower after handwashing, but neither paired comparison was statistically significant after Holm adjustment. Individual responses varied, with CFU counts decreasing and increasing after handwashing in some participants. These findings do not provide statistically significant evidence of a reduction in culturable bacterial bioburden under the conditions assessed.

Left- and right-hand measurements were analysed separately to describe changes for each anatomical hand. Although each analysis accounted for the pairing of pre- and post-handwashing measurements within participants, the analyses did not explicitly model the correlation between hands. Holm adjustment addressed multiplicity across the microbiological comparisons, including both hand-specific CFU tests. However, separate within-hand comparisons do not establish whether the magnitude of change differs between hands, as no direct comparison of these changes was performed. Hand dominance was not recorded; therefore, the findings cannot be attributed to dominant or non-dominant hand use.

Both microbiological sampling points occurred during the same follow-up visit. Consequently, the findings concern the immediate pre- and post-handwashing comparison rather than microbiological changes over the three months following education. The absence of a pre-education microbiological baseline and a control group prevents attribution of the observed differences to the educational intervention or conclusions about its sustained effectiveness.

Previous studies have reported reductions in hand bacterial contamination following handwashing [25,26,27]. However, bacterial recovery after washing can vary with technique, duration, friction, and other procedural conditions [28,29]. At the present follow-up visit, soap use was monitored and clean running water was available, but handwashing duration was not monitored or recorded. Variation in handwashing performance may therefore have contributed to the observed findings, although its contribution was not established.

Although teachers were asked to provide periodic monitoring and positive reinforcement during the three-month interval, this support does not establish that students consistently followed the recommended handwashing practices in their daily routines. No structured behavioural or adherence measurements were collected; therefore, adherence during this interval was an unmeasured factor. Previous studies have highlighted the importance of continued reinforcement, reminders, and environmental support in maintaining hand hygiene behaviours [30,31]. However, declining adherence or loss of previously taught skills cannot be inferred from the present data or used to explain the microbiological findings. Future evaluations should incorporate repeated behavioural assessments alongside microbiological sampling.

Incomplete washing of areas such as fingertips, nail margins, and skin folds may limit bacterial removal [32,33]. Although participants had previously received instruction on handwashing techniques, the present study did not establish whether incomplete coverage explained the observed microbiological findings. This remains a possible explanation rather than a demonstrated mechanism.

Culturable bacterial bioburden and organism-specific detection represent different endpoints. CFU counts quantify colonies recovered from each hand imprint under the specified culture conditions, whereas organism-specific detection records whether a particular organism was recovered from a participant, irrespective of its abundance. A decrease in the detection frequency of selected organisms therefore need not produce a comparable reduction in overall bacterial counts, which also include colonies of other recovered bacteria [34]. Moreover, colony selection for identification was morphology-guided rather than exhaustive, and species-specific contributions to the total CFU count were not quantified. This distinction provides a possible explanation for the different patterns across endpoints, but the underlying changes in bacterial composition were not established quantitatively. Neither endpoint establishes persistent colonisation or the sustained effectiveness of the earlier educational intervention.

### 4.2. Incidental Recovery of Yeast and Mould Before and After Handwashing

Yeast and mould were incidentally recovered from the blood agar hand imprint cultures. Detection frequencies were higher after handwashing, but none of the paired comparisons was statistically significant after Holm adjustment. These findings describe exploratory observations under the culture conditions used and do not establish that handwashing increased fungal contamination or that the earlier educational intervention was ineffective.

Blood agar incubation at 37 °C for 18–24 h was intended for bacterial recovery and was not optimised for fungal detection. Dedicated mould identification procedures use fungal media and longer growth periods; for example, the CDC describes using Sabouraud dextrose agar and cultures grown for several days before identification [35]. The present protocol may therefore underrepresent fungi requiring different culture conditions or longer incubation. Consequently, the reported detection frequencies should not be interpreted as a comprehensive assessment of fungal burden or diversity, and non-detection does not establish the absence of fungi.

Previous research has documented fungi in air and deposited dust within primary school environments [36,37]. Exposure to shared handwashing facilities or surrounding surfaces may contribute to fungal transfer after washing [38]. These findings provide context for environmental exposure as a possible contributor to incidental fungal recovery, but they do not identify the source of the fungi recovered in the present study.

Participants used tissue paper to dry their hands before post-handwashing sampling. Drying time and residual hand moisture were not monitored to let them practice on their own what they learnt in terms of educational intervention. Contamination of drying materials and environmental contact immediately before sampling were not evaluated as sources of the recovered fungi. Residual moisture [39,40,41], environmental exposure, and sampling variability [36,42] therefore remain hypotheses that could be investigated in future studies; their contributions to the non-significant increases in yeast and mould recovery were not established.

Contamination of the handwashing water or water-storage system is another possible source that cannot be evaluated from hand imprint cultures alone. The present findings do not establish tank water as the source of the recovered fungi. Future investigations should incorporate dedicated fungal culture and identification, together with contemporaneous sampling of the handwashing water, any storage tanks supplying it, and relevant drying materials or contact surfaces. Such assessments would help distinguish incidental recovery from contamination associated with the handwashing and sampling procedures.

### 4.3. Bacterial Detection and Species Distribution Following Handwashing

Lower recovery frequencies of selected bacteria were observed immediately after handwashing at the three-month follow-up visit. These findings describe immediate changes in culturable hand contamination among children who had previously received hand hygiene education. Previous studies have also documented bacterial contamination of schoolchildren’s hands, highlighting the relevance of hand hygiene in school settings [10,43]. Reduced recoverability of selected organisms may indicate fewer opportunities for exposure; however, exposure and bacterial transmission were not measured in the present study. The findings therefore do not demonstrate reduced transmission or establish that the educational intervention caused the observed differences or produced sustained benefits.

After Holm adjustment, only the reduction in *E. coli*/*Shigella* complex detection remained statistically significant, decreasing from 21/60 participants (35.0%) before handwashing to none afterwards (adjusted *p* < 0.001). Neither hand-specific CFU comparison was statistically significant after adjustment. Accordingly, the findings should not be interpreted as demonstrating significant reductions across Gram-positive and Gram-negative bacteria generally. The change in detection of this bacterial complex and the non-significant CFU comparisons address different outcomes and are not inherently contradictory.

Gram-positive bacteria, particularly *Staphylococcus* spp., were remained frequently recovered after handwashing, consistent with previous reports of staphylococci on human skin and hands [44,45,46,47]. *Staphylococcus aureus* and *Staphylococcus saprophyticus* were recovered from fewer participants after washing. Although staphylococci can persist within the skin microenvironment [48], recovery from these hand imprints cannot determine whether an organism represented resident microbiota or transient contamination. The findings therefore describe culture positivity rather than established carriage, and they do not establish a reduction in infection risk.

Several Gram-negative organisms, including *E. coli*/*Shigella* complex, *Klebsiella pneumoniae*, *Enterobacter cloacae*, *Stenotrophomonas maltophilia*, and *Pseudomonas aeruginosa*, were recovered less frequently or were no longer detected after handwashing. Environmental or faecal contamination is a recognised source of bacteria recovered from hands [49], although the sources of organisms in this study were not determined. Non-detection after washing does not establish eradication or sustained absence. Biofilm formation has been described as a persistence mechanism in *Pseudomonas aeruginosa* and *Acinetobacter baumannii* [50,51]; however, its contribution to recovery in this study was not investigated.

Some organisms were detected only after handwashing. Such findings should not automatically be interpreted as acquisition caused by washing. Variation in bacterial transfer during the blood agar imprint procedure [52,53] and selective recovery by culture-based methods [53] may contribute to differences between samples. The present study could not distinguish these explanations from environmental exposure or other sources of variation.

Overall, these findings describe immediate differences in the detection of culturable surface-associated bacteria. They do not establish persistent colonisation, changes in bacterial carriage over time, or sustained improvements attributable to the educational intervention.

### 4.4. Antimicrobial Resistance Patterns Among Hand-Associated Staphylococcus spp.

The antimicrobial susceptibility and genomic analyses characterised selected recovered isolates. Antimicrobial susceptibility testing revealed diverse resistance patterns among hand-associated *Staphylococcus* spp. Resistance to fusidic acid, penicillin and erythromycin was frequently observed within the tested collection, whereas all isolates were susceptible to linezolid and most were susceptible to rifampicin. These findings demonstrate the recovery of antimicrobial-resistant staphylococci from the hand surfaces of apparently healthy Aboriginal schoolchildren, supporting further investigation of AMR in community settings. Thirteen of the 20 *S. arlettae* isolates (65.0%) met the adapted MDR definition, including all four selected for whole-genome sequencing. Their recovery documents their presence at sampling but does not establish infection, persistent colonisation or transmission.

Previous studies have demonstrated that staphylococcal carriage and antimicrobial resistance occur among community and Indigenous populations. Among Orang Asli communities in northeastern Peninsular Malaysia, *S. aureus* carriage was reported in 47.2% of participants aged 18–65 years [54]. Methicillin-resistant *Staphylococcus aureus* (MRSA) nasal carriage has also been investigated among Malaysian students [55], while antimicrobial-resistant *S. aureus* has been recovered from rural communities in Sarawak [56]. Studies among Indigenous populations elsewhere, including Brazilian communities, have reported *S. aureus* and community-associated MRSA carriage [57]. These studies provide context for investigating antimicrobial-resistant staphylococci outside healthcare settings. However, differences in sampling sites, participant characteristics and isolate selection limit direct comparisons with the present hand-imprint findings.

Penicillin resistance was particularly frequent among *S. arlettae* and *S. aureus*, affecting 20/20 and 7/8 isolates, respectively. Resistance was also observed in 2/6 *S. haemolyticus* and 2/5 *S. pasteuri* isolates. Previous community studies have likewise reported penicillin resistance among *S. aureus* isolates [54,56]. In contrast, all *S. aureus* isolates were susceptible to cefoxitin, indicating a methicillin-susceptible *Staphylococcus aureus* (MSSA), consistent with previous findings among Aboriginal and other Indigenous populations [54,56,58]. Cefoxitin resistance was detected in one *S. arlettae* and one *S. haemolyticus* isolate, whereas all *S. saprophyticus* isolates were susceptible. These findings distinguish penicillin resistance from the cefoxitin phenotype used to screen for methicillin resistance; the phenotypic results alone do not establish the underlying mechanisms.

Fusidic acid resistance was frequently observed among *S. saprophyticus*, *S. ureilyticus* and *S. arlettae*, consistent with previous reports of resistant skin-associated staphylococci recovered from healthy individuals [59,60]. Erythromycin resistance was also detected among several CoNS species, consistent with previous community-based studies [60,61,62]. Clindamycin resistance was uncommon, but intermediate susceptibility occurred in 15/20 *S. arlettae* isolates (75.0%). These intermediate results contributed to MDR classification as non-susceptible findings while remaining separately identified in the AST results.

Novobiocin findings were considered separately. All tested *S. saprophyticus*, *S. arlettae* and *S. ureilyticus* isolates were categorised as resistant. Intrinsic novobiocin resistance has been demonstrated in *S. saprophyticus* [23,61,63,64], but the mechanisms responsible for the observed phenotypes were not investigated in the present isolates. Novobiocin results were therefore reported descriptively, excluded from MDR classification and not interpreted as evidence of clinically relevant acquired resistance or acquisition of resistance within the study community. Both novobiocin and penicillin G were excluded from the MDR category count. Novobiocin was excluded because of its limited contemporary therapeutic relevance and predictable intrinsic resistance in some Staphylococcus species, whereas penicillin G was excluded because penicillin resistance could not be interpreted consistently as acquired resistance across all tested Staphylococcus species.

All tested isolates were susceptible to linezolid. For rifampicin, one *S. ureilyticus* isolate was intermediate and the remaining isolates were susceptible; no resistant isolates were detected. These findings describe the tested collection and do not establish that resistance to these agents is uncommon throughout the community. Figure 2 illustrates similarities and differences in susceptibility profiles within and between species. These phenotypic patterns support further investigation of resistance determinants but do not establish genetic relatedness, persistence or transmission.

The antimicrobial susceptibility findings describe all recovered pre-handwashing isolates belonging to six purposively selected species. Selection at the species level limits the generalisability of the observed percentages. These findings should therefore be interpreted as characteristics of the tested isolates rather than estimates of resistance prevalence among the schoolchildren or the complete collection of hand-associated staphylococci. The study also did not assess antibiotic prescribing or consumption in the community, so their contribution to the observed resistance patterns remains unknown.

### 4.5. Antimicrobial Resistance Genes and Genotype–Phenotype Concordance in Staphylococcus arlettae

Partial genotype–phenotype concordance was an important finding in the four sequenced *S. arlettae* isolates. Detection of *msr(A)* and, in three isolates, *mph(C)* was consistent with the erythromycin-resistant phenotype, while *dfrG* was consistent with resistance to the trimethoprim component of trimethoprim/sulfamethoxazole. However, ResFinder detected no corresponding acquired resistance determinants for penicillin and fusidic acid resistance or for intermediate susceptibility to clindamycin in these four isolates. The detected genes therefore accounted for only part of the observed antimicrobial susceptibility profile.

Two matches spanning the full reference sequence were reported for *msr(A)* in isolate 508R(2), with nucleotide identities of 86.78% and 99.05% to their respective database references. These percentages do not represent the pairwise identity between the two recovered sequences. The findings could reflect distinct *msr(A)*-related loci, potentially including paralogous copies, but assembly-related duplication or sequence errors cannot be excluded. Distinguishing these possibilities requires examination of the genomic locations, flanking sequences and read support. Therefore, they were interpreted as two putative *msr(A)*-related sequence matches rather than confirmed functional gene copies.

The concurrent detection of *msr(A)* and *mph(C)* in three isolates indicates the presence of multiple macrolide resistance determinants. The *msr(A)* gene encodes an ATP-binding cassette (ABC-F) ribosomal protection protein, whereas *mph(C)* encodes a macrolide phosphotransferase that enzymatically inactivates macrolides through phosphorylation [65,66]. Previous studies have documented co-occurrence and physical linkage of *msr(A)* and *mph(C)* among staphylococci, including their association on the same plasmid in *S. aureus* [67,68]. However, physical linkage, gene expression and the combined contribution of these determinants to the resistance phenotype were not established in the present *S. arlettae* isolates. In isolate 603R(3), *msr(A)* was detected in the absence of *mph(C)*, which was consistent with its erythromycin-resistant phenotype, although the relative contribution of individual resistance mechanisms was not determined.

The presence of *dfrG* in all four isolates indicates acquired resistance to trimethoprim through the production of an alternative dihydrofolate reductase with reduced affinity for the antimicrobial agent [69]. The *dfrG* gene is one of the most prevalent trimethoprim resistance determinants reported in both *Staphylococcus aureus* and coagulase-negative staphylococci and has been identified in clinical, livestock, and community-associated isolates, highlighting its widespread dissemination among staphylococci [69,70,71,72].

Although high genotype–phenotype concordance has been reported in other bacterial collections [73], the interpretation of WGS-based antimicrobial susceptibility predictions requires consideration of the available resistance knowledge and validation for the organism–antimicrobial combination [74]. An inter-laboratory study also demonstrated that bioinformatic analyses of the same sequence datasets can yield differing resistance predictions [75]. These findings support cautious interpretation of acquired resistance gene screening in a less-characterised species such as *S. arlettae*.

ResFinder results depend on database content and analysis settings, and an acquired-gene search alone does not comprehensively assess chromosomal resistance mechanisms. Although the ResFinder framework can incorporate PointFinder searches for supported species, the ResFinder 4.0 validation included *S. aureus* rather than *S. arlettae*. Its reported predictive performance should therefore not be assumed to apply unchanged to the latter species. Potential explanations for discordance include chromosomal mutations, intrinsic mechanisms, altered gene expression and uncharacterised or divergent resistance determinants; these mechanisms were not established in our isolates [76].

Accordingly, absence of a ResFinder match should not be interpreted as absence of a genetic resistance mechanism or evidence of susceptibility. The genomic findings should be interpreted alongside phenotypic AST. Further investigation of the discordant isolates, including verification of phenotypic classifications, complementary database searches and targeted examination of candidate chromosomal variants, would help clarify the underlying mechanisms.

### 4.6. Virulence-Associated Genomic Features

Whole-genome analysis revealed a limited virulence-associated profile among the four *Staphylococcus arlettae* isolates. VirulenceFinder did not identify major staphylococcal toxin genes, including staphylococcal enterotoxins, toxic shock syndrome toxin-1 (TSST-1), exfoliative toxins, Panton–Valentine leukocidin (PVL), or other classical determinants associated with invasive staphylococcal infections. The absence of these virulence factors is consistent with the generally recognised behaviour of CoNS, including *S. arlettae*, which are predominantly regarded as commensal organisms associated with skin colonisation and environmental persistence rather than highly pathogenic species [64,77]. Nevertheless, some CoNS can act as opportunistic pathogens, particularly in healthcare-associated infections, where pathogenicity is often influenced by antimicrobial resistance, biofilm formation, and host susceptibility rather than toxin production alone [14,77].

VirulenceFinder identified ACME-associated sequences in all four *S. arlettae* isolates, with seven to nine detected hits per isolate and nucleotide identities ranging from approximately 78–89%. The relatively low sequence identities suggest that these findings may represent partial homologous regions related to ACME-associated sequences rather than confirmation of a complete functional ACME element. ACME is best characterised in *Staphylococcus epidermidis* and epidemic community-associated methicillin-resistant *Staphylococcus aureus* (MRSA) USA300, where it contributes primarily to bacterial fitness, adaptation, and colonisation rather than direct toxin-mediated virulence [18,78]. This mobile genetic element contains functional components, including the arginine deiminase pathway (*arc* operon), which may enhance survival under acidic stress, and the oligopeptide transport system (*opp3* operon), which may contribute to bacterial fitness and colonisation [79]. Therefore, ACME is considered a colonisation- and survival-associated mobile genetic element rather than a classical virulence determinant, and its presence alone does not necessarily indicate increased invasive pathogenic potential [79,80,81,82].

The partial ACME-associated sequence matches provide additional information on the genomic characteristics of these selected *S. arlettae* isolates. However, these matches do not establish the presence of a complete functional ACME element or its contribution to bacterial adaptation and persistence. Biofilm- and adhesion-associated genes were not systematically analysed, and the detected sequences do not demonstrate biofilm formation, adhesion capacity or persistence on the children’s hands. Further genomic and functional characterisation would be required to determine the biological significance of these findings.

### 4.7. Plasmid Replicon Characterisation

The identification of a RepA_N-family replicon in isolates 208R and 508R(2) indicates the presence of plasmid-associated replication machinery among *Staphylococcus arlettae* isolates. RepA_N represents the N-terminal domain of plasmid replication initiation proteins involved in plasmid replication and maintenance, thereby enabling the stable inheritance of plasmid DNA during bacterial cell division [83]. The detection of the rep24c replicon in isolates 208R and 508R(2) indicates the presence of a RepA_N-family plasmid associated with these replication and maintenance functions. The presence of the same rep24c replicon in two isolates suggests that these strains may harbour related plasmid backbones or share a common plasmid lineage. Further plasmid characterisation, such as complete plasmid assembly and comparative plasmid analysis, would be required to determine the genetic content and potential contribution of these plasmids to antimicrobial resistance dissemination in *S. arlettae* [84].

### 4.8. Pathogen Prediction

PathogenFinder2 assigned all four *S. arlettae* isolates to its “Human Non-Pathogenic” category; this computational classification does not establish their inability to cause human infection. The relatively low prediction scores (0.3259–0.4538) indicate limited similarity between the predicted protein repertoires of these isolates and those of recognised human pathogens included in the PathogenFinder2 training dataset. These findings are consistent with the established ecology of *S. arlettae*, which is predominantly regarded as a commensal CoNS species recovered from animal-associated environments, environmental sources, and occasionally healthy human skin [70]. Although *S. arlettae* is considered an opportunistic pathogen rather than a primary pathogen, infections have been reported under specific clinical conditions, supporting its classification as a low-virulence organism with opportunistic potential [85].

All four isolates received the “Human Non-Pathogenic” classification from PathogenFinder2, while VirulenceFinder screening reported partial ACME-associated matches without detected classical staphylococcal toxin genes. Although ACME has been implicated in bacterial fitness and colonisation in *S. aureus* USA300 [79,82], the partial ACME-associated matches identified in our *S. arlettae* isolates do not establish a complete functional element or its biological contribution. These computational findings provide preliminary information on selected genomic features but do not demonstrate adaptation to human skin, environmental persistence, reduced virulence or inability to cause infection.

PathogenFinder2 predictions provide an estimation of pathogenic potential based on genomic similarity to known human pathogens and do not directly determine the ability of an organism to cause infection. Although infections caused by *S. arlettae* are uncommon, sporadic cases of bacteraemia, prosthetic joint infection, and catheter-associated infection have been reported, particularly among immunocompromised individuals and patients with implanted medical devices [85]. These observations indicate that disease caused by *S. arlettae* is likely influenced by host susceptibility and clinical circumstances rather than intrinsic virulence alone [14,70].

CoNS, including *S. arlettae*, commonly colonise human skin and may become opportunistic pathogens when normal skin barriers are disrupted or host defence mechanisms are compromised. Although Aboriginal schoolchildren are generally healthy individuals and do not share the clinical risk factors of immunocompromised patients, their hands represent a site of continuous interaction between human skin microbiota and the surrounding environment. The recovery of diverse *Staphylococcus* spp. demonstrates their presence on the sampled hand surfaces. In particular, detection of *S. arlettae* does not establish active infection, persistent colonisation, or its long-term presence within the participants’ skin microbiota.

Collectively, the WGS analyses identified selected acquired antimicrobial resistance determinants and partial virulence-associated sequence matches in four *S. arlettae* isolates. These findings provide preliminary genomic characterisation but do not establish pathogenicity, persistent colonisation or transmission.

### 4.9. Limitations

This study had several limitations. The microbiological assessment comprised paired samples collected immediately before and after a single handwashing episode at one follow-up visit. There was no pre-education microbiological baseline or control arm as this was a community service program.

Accordingly, these findings reflect immediate, within-participant responses measured under controlled experimental conditions. To isolate baseline sampling variables and assess long-term behavioural persistence, future multi-centre trials incorporating parallel control cohorts will serve as a natural extension of this groundwork.

Sufficient estimates of means and standard deviations were unavailable from previous studies; therefore, the original sample-size calculation assumed a standardised paired effect size of d_z_ = 0.40 for a two-sided paired *t*-test and did not designate a single primary endpoint. Although the 60 complete pairs exceeded the 52 required under those assumptions, the calculation did not establish power for the individual Wilcoxon signed-rank or McNemar comparisons or for multiplicity-adjusted analyses. The sample may therefore have limited the ability to detect modest changes, particularly after Holm adjustment. Nevertheless, non-significant findings do not establish the absence of an effect, and their cause cannot be attributed solely to sample size.

Handwashing duration and everyday adherence during the three-month interval were not systematically measured. Although teachers were asked to provide periodic monitoring and positive reinforcement after the handwashing training, this practice was not observed by researchers. Left- and right-hand outcomes were analysed separately, and their correlation was not explicitly modelled. Holm adjustment addressed multiple testing but did not provide a direct comparison of changes between hands.

The hand imprint method measures microorganisms transferred from sampled hand surfaces and recoverable under the culture conditions used. It does not quantify the complete hand microbiota or distinguish resident microbiota from transient contamination. Recovery may vary with contact area, pressure, residual moisture and sampling variability. Findings therefore concern culturable surface contamination and do not establish persistent colonisation, exposure or transmission.

Yeast and mould were incidental findings under culture conditions intended for bacterial recovery. The absence of a dedicated fungal culture protocol limits the sensitivity and scope of these observations. Consequently, the reported detection frequencies cannot establish the overall extent of fungal contamination, and potential contributions from handwashing water or drying materials were not established.

Colony selection was morphology-guided and targeted selected bacterial groups rather than exhaustively sampling all colonies. Consequently, some organisms, including those with similar colony appearances, may have been missed, and the reported species distribution reflects the isolates recovered through this selection procedure.

Species identification was limited by the resolution and reference-database coverage of MALDI-TOF MS. Routine MALDI-TOF MS does not reliably distinguish *Shigella* spp. from *Escherichia coli* [86]. The additional culture and TSI, urea and SIM findings did not establish a definitive species-level assignment for the affected isolates, which were therefore reported as *E. coli*/*Shigella* complex. Their recovery should not be interpreted as confirmed *S. sonnei* detection or evidence of a specific pathogenic *E. coli* type.

Antimicrobial susceptibility testing covered all recovered pre-handwashing isolates belonging to six purposively selected species, and WGS was restricted to four of the 13 MDR *S. arlettae* isolates. Four of the six species had fewer than 10 tested isolates, limiting the precision of the species-specific susceptibility percentages. The four sequenced isolates were selected on the basis of their phenotypic resistance profiles and cannot represent the full genomic diversity of the recovered *S. arlettae* collection. These findings should not be generalised to all hand-associated staphylococci or used as participant-level resistance prevalence estimates.

The assemblies were polished using two rounds of Medaka with Oxford Nanopore reads only, without short-read polishing. Residual insertion and deletion errors, particularly in homopolymeric regions, may remain after polishing [87]. Within coding sequences, such errors can introduce apparent frameshifts or premature stop codons, potentially affecting predicted gene integrity, sequence identity and downstream gene detection. Consequently, apparent gene truncation or an absent database match should not be interpreted as definitive evidence of biological gene disruption or absence without further validation. In addition, database-dependent genomic screening cannot establish the absence of unrecognised resistance or virulence determinants. Biofilm- and adhesion-associated genes were not systematically analysed, and predicted genomic features were not evidence of their expression or biological activity.

## 5. Conclusions

Among 60 Orang Asli schoolchildren, neither hand-specific CFU comparison was statistically significant after Holm adjustment, although *E. coli*/*Shigella* complex recovery decreased immediately after handwashing (Holm-adjusted *p* < 0.001). These findings describe a single follow-up assessment and do not establish the effectiveness of the earlier educational intervention. AST characterised 71 pre-handwashing isolates from six selected *Staphylococcus* species, and sequencing of four MDR *S. arlettae* isolates identified resistance determinants and other genomic features with partial genotype–phenotype concordance. These selected-isolate findings do not establish community resistance prevalence, persistent colonisation or transmission. Future studies should incorporate a pre-education microbiological baseline, a comparison group and repeated microbiological and behavioural assessments to evaluate sustained outcomes.

## Figures and Tables

**Figure 1:**
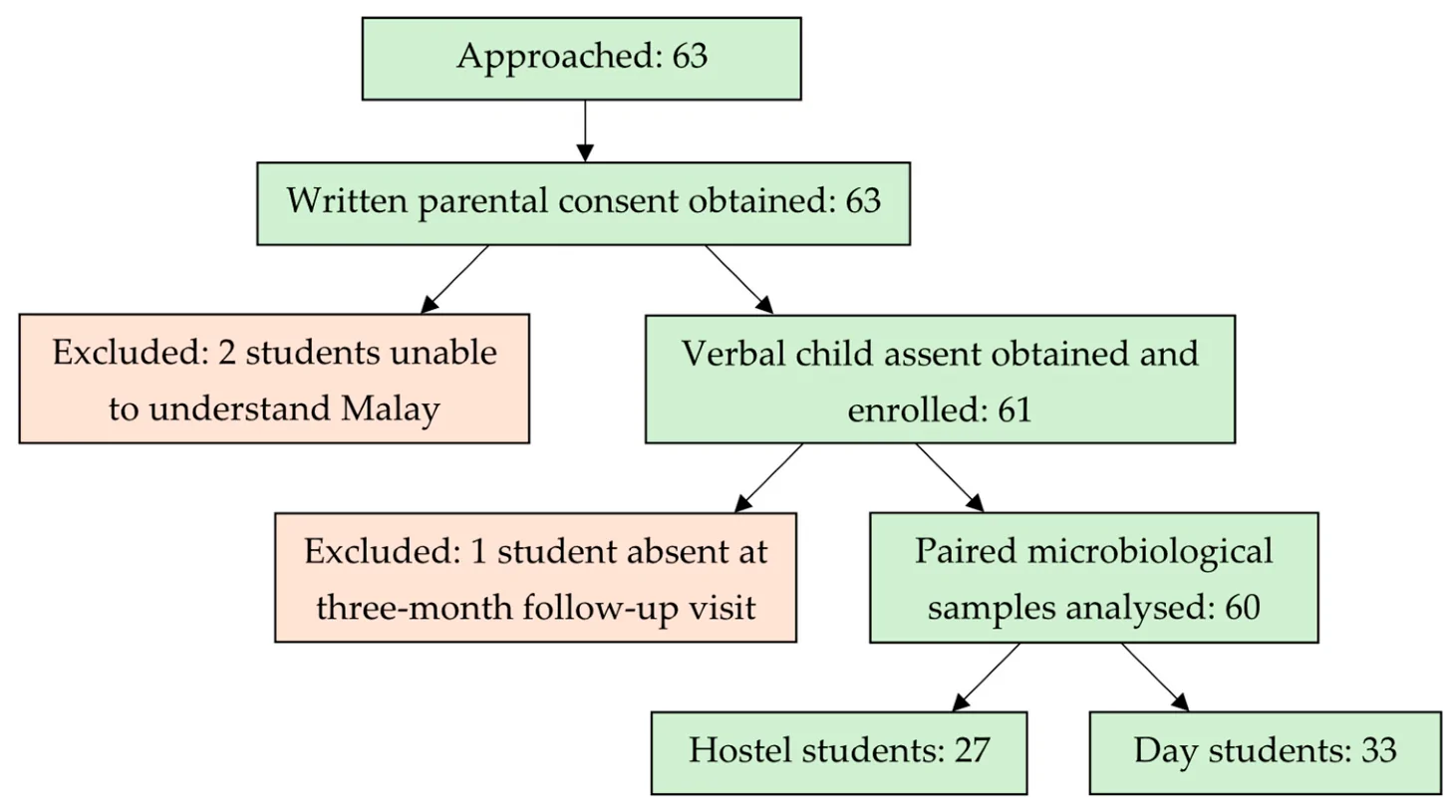
Participant recruitment and inclusion in the paired microbiological analysis at the three-month follow-up visit.

**Figure 2:**
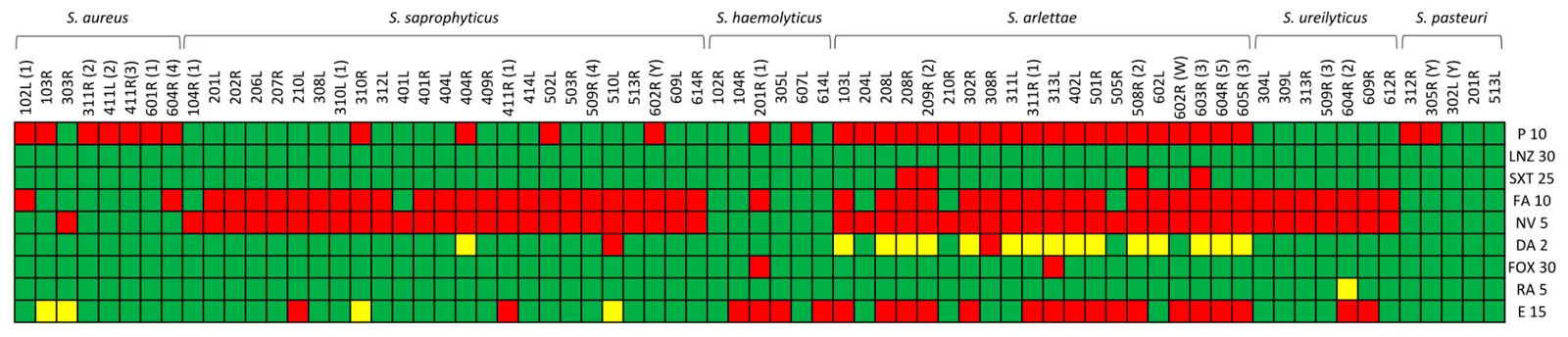
Heatmap showing isolate-level antimicrobial susceptibility patterns of selected *Staphylococcus* spp. Green indicates susceptible, yellow indicates intermediate, and red indicates resistant phenotypes.

**Table 1:** Comparison of culturable bacterial bioburden (log_10_ CFUs/plate) before and after handwashing (*n* = 60).

Hand	Before	After	Test Statistic	Unadjusted *p*-Value	Holm-Adjusted *p*-Value
Left	2.5668 (0.2212) ^a^	2.4624 (0.3138) ^a^	t(59) = −2.379	0.021 ^1^	1.000
Right	2.5508 (2.3881–2.6964) ^b^	2.5125 (2.3071–2.7092) ^b^	Z = −1.612	0.107 ^2^	1.000

^a^ Mean (SD). ^b^ Median (Q1–Q3). ^1^ Paired-samples *t*-test. ^2^ Wilcoxon signed-rank test; asymptotic two-sided *p*-value. Note: Values are expressed as log_10_ CFUs/plate. Paired differences were defined as post- minus pre-handwashing values. Unadjusted two-sided *p*-values were obtained from the corresponding paired statistical tests. Holm-adjusted *p*-values were calculated across 52 microbiological comparisons. Statistical significance was assessed using adjusted *p*-values < 0.05.

**Table 2:** Frequency of incidental yeast and mould recovery from blood agar hand imprint cultures immediately before and after handwashing at the three-month follow-up visit (*n* = 60).

Organism	Hand	Before, *n* (%)	After, *n* (%)	Unadjusted *p*-Value	Holm-Adjusted *p*-Value
Yeast	Left	9 (15.0)	12 (20.0)	0.629	1.000
	Right	11 (18.3)	18 (30.0)	0.189	1.000
Mould	Left	29 (48.3)	38 (63.3)	0.163	1.000
	Right	34 (56.7)	37 (61.7)	0.701	1.000

Note: Data are presented as *n* (%) of participants with yeast or mould detected on the indicated hand. Paired pre- and post-handwashing comparisons were analysed using two-sided exact McNemar tests. Both unadjusted and Holm-adjusted two-sided *p*-values are reported. Holm adjustment was applied across 52 microbiological comparisons, with adjusted *p*-values < 0.05 considered statistically significant. These exploratory findings reflect recovery under bacterial culture conditions and do not represent a comprehensive assessment of fungal contamination.

**Table 3:** Frequency of participants with hand cultures positive for Gram-positive and Gram-negative bacteria immediately before and after handwashing at the three-month follow-up visit (*n* = 60).

Gram Category	Before, *n* (%)	After, *n* (%)	Unadjusted *p*-Value	Holm-Adjusted *p*-Value
Gram-positive organisms	58 (96.70)	50 (83.30)	0.008	0.391
Gram-negative organisms	40 (66.67)	27 (45.00)	0.019	0.939

Note: Data are presented as n (%) of participants. A participant was classified as positive for a Gram category if at least one bacterium from that category was recovered from either hand at the corresponding sampling point. Each participant was counted once per category per sampling point. Both Gram categories could be detected in the same participant; therefore, the categories were not mutually exclusive. Paired comparisons were analysed using two-sided exact McNemar tests. Both unadjusted and Holm-adjusted two-sided *p*-values are reported. Holm adjustment was applied across 52 microbiological comparisons, with adjusted *p*-values < 0.05 considered statistically significant.

**Table 4:** Frequency of participants with hand cultures positive for each bacterial taxon immediately before and after handwashing at the three-month follow-up visit (*n* = 60).

Possible Pathogens	Before, *n* (%)	After, *n* (%)	Unadjusted *p*-Value ^1^	Holm-Adjusted *p*-Value
Gram-positive organisms				
*Staphylococcus aureus*	7 (11.67)	1 (1.67)	0.031	1.000
*Staphylococcus saprophyticus*	22 (36.67)	13 (21.67)	0.004	0.199
*Staphylococcus arlettae*	18 (30.00)	12 (20.00)	0.031	1.000
*Staphylococcus ureilyticus*	9 (15.00)	6 (10.00)	0.375	1.000
*Staphylococcus haemolyticus*	6 (10.00)	1 (1.67)	0.063	1.000
*Staphylococcus pasteuri*	5 (8.33)	5 (8.33)	1.000	1.000
*Staphylococcus epidermidis*	5 (8.33)	2 (3.33)	0.250	1.000
*Staphylococcus schweitzeri*	1 (1.67)	0 (0.00)	1.000	1.000
*Staphylococcus sciuri*	4 (6.67)	2 (3.33)	0.500	1.000
*Staphylococcus cohnii*	2 (3.33)	1 (1.67)	1.000	1.000
*Staphylococcus kloosii*	7 (11.67)	6 (10.00)	1.000	1.000
*Staphylococcus caprae*	1 (1.67)	1 (1.67)	1.000	1.000
*Staphylococcus lutrae*	3 (5.00)	1 (1.67)	0.500	1.000
*Staphylococcus hominis*	6 (10.00)	7 (11.67)	1.000	1.000
*Staphylococcus nepalensis*	5 (8.33)	3 (5.00)	0.500	1.000
*Staphylococcus capitis*	1 (1.67)	1 (1.67)	1.000	1.000
*Staphylococcus borealis*	1 (1.67)	1 (1.67)	1.000	1.000
*Staphylococcus muscae*	1 (1.67)	1 (1.67)	1.000	1.000
*Staphylococcus equorum*	2 (3.33)	1 (1.67)	1.000	1.000
*Staphylococcus carnosus*	2 (3.33)	2 (3.33)	1.000	1.000
*Staphylococcus warneri*	2 (3.33)	4 (6.67)	0.625	1.000
*Staphylococcus xylosus*	3 (5.00)	2 (3.33)	1.000	1.000
*Staphylococcus* sp.	0 (0.00)	4 (6.67)	0.125	1.000
Gram-negative organisms				
*E. coli*/*Shigella* complex	21 (35.00)	0 (0.00)	<0.001	<0.001
*Klebsiella pneumoniae*	6 (10.00)	0 (0.00)	0.031	1.000
*Acinetobacter variabilis*	8 (13.33)	5 (8.33)	0.549	1.000
*Acinetobacter indicus*	2 (3.33)	1 (1.67)	1.000	1.000
*Acinetobacter baumannii*	6 (10.00)	1 (1.67)	0.125	1.000
*Acinetobacter ursingii*	1 (1.67)	0 (0.00)	1.000	1.000
*Acinetobacter schindleri*	1 (1.67)	4 (6.67)	0.375	1.000
*Acinetobacter nosocomialis*	1 (1.67)	1 (1.67)	1.000	1.000
*Acinetobacter radioresistens*	1 (1.67)	0 (0.00)	1.000	1.000
*Acinetobacter* sp.	1 (1.67)	0 (0.00)	1.000	1.000
*Enterobacter cloacae*	2 (3.33)	0 (0.00)	0.500	1.000
*Moraxella osloensis*	11 (18.33)	16 (26.67)	0.383	1.000
*Massilia varians*	1 (1.67)	2 (3.33)	1.000	1.000
*Massilia timonae*	0 (0.00)	1 (1.67)	1.000	1.000
*Stenotrophomonas maltophilia*	2 (3.33)	0 (0.00)	0.500	1.000
*Pseudomonas aeruginosa*	1 (1.67)	0 (0.00)	1.000	1.000
*Pseudomonas oryzihabitans*	1 (1.67)	0 (0.00)	1.000	1.000
*Pseudomonas stutzeri*	0 (0.00)	1 (1.67)	1.000	1.000
*Pseudomonas* sp.	0 (0.00)	1 (1.67)	1.000	1.000
*Brevundimonas diminuta*	0 (0.00)	1 (1.67)	1.000	1.000
*Yersinia enterocolitica*	1 (1.67)	0 (0.00)	1.000	1.000

^1^ McNemar test. Note: Data are presented as *n* (%) of participants. Each participant was counted once per bacterial taxon at each sampling point if that taxon was recovered from either hand, regardless of the number of isolates recovered. Paired pre- and post-handwashing comparisons were analysed using two-sided exact McNemar tests. Both unadjusted and Holm-adjusted *p*-values are reported. Holm–Bonferroni correction was applied across 52 microbiological comparisons, with adjusted *p*-values < 0.05 considered statistically significant. Discordant-pair counts and paired changes in detection proportion with unadjusted 95% confidence intervals are provided in Supplementary File S2. *E. coli*/*Shigella* complex comprises isolates initially identified presumptively as *S. sonnei* by MALDI-TOF MS and biochemical tests, but for which definitive species-level identification was not established.

**Table 5:** Antimicrobial susceptibility profiles of selected pre-handwashing *Staphylococcus* isolates.

Organisms (*n*)	Susceptibility Patterns	P 10 ^a^	LNZ 30	SXT 25	FA 10 ^b^	NV 5	DA 2	FOX 30 ^a^	RA 5	E 15
*S. aureus* (8)	Susceptible, *n* (%)	1 (12.50)	8 (100.00)	8 (100.00)	6 (75.00)	7 (87.50)	8 (100.00)	8 (100.00)	8 (100.00)	6 (75.00)
	Intermediate, *n* (%)	N/A	0 (0.00)	0 (0.00)	N/A	0 (0.00)	0 (0.00)	N/A	0 (0.00)	2 (25.00)
	Resistant, n (%)	7 (87.50)	0 (0.00)	0 (0.00)	2 (25.00)	1 (12.50)	0 (0.00)	0 (0.00)	0 (0.00)	0 (0.00)
*S. saprophyticus* (25)	Susceptible, *n* (%)	21 (84.00)	25 (100.00)	25 (100.00)	2 (8.00)	0 (0.00)	23 (92.00)	25 (100.00)	25 (100.00)	21 (84.00)
	Intermediate, *n* (%)	N/A	0 (0.00)	0 (0.00)	N/A	0 (0.00)	1 (4.00)	N/A	0 (0.00)	2 (8.00)
	Resistant, *n* (%)	4 (16.00)	0 (0.00)	0 (0.00)	23 (92.00)	25 (100.00)	1 (4.00)	0 (0.00)	0 (0.00)	2 (8.00)
*S. ureilyticus* (7)	Susceptible, *n* (%)	7 (100.00)	7 (100.00)	7 (100.00)	0 (0.00)	0 (0.00)	7 (100.00)	7 (100.00)	6 (85.71)	5 (71.43)
	Intermediate, *n* (%)	N/A	0 (0.00)	0 (0.00)	N/A	0 (0.00)	0 (0.00)	N/A	1 (14.29)	0 (0.00)
	Resistant, *n* (%)	0 (0.00)	0 (0.00)	0 (0.00)	7 (100.00)	7 (100.00)	0 (0.00)	0 (0.00)	0 (0.00)	2 (28.57)
*S. arlettae* (20)	Susceptible, *n* (%)	0 (0.00)	20 (100.00)	16 (80.00)	3 (15.00)	0 (0.00)	4 (20.00)	19 (95.00)	20 (100.00)	5 (25.00)
	Intermediate, *n* (%)	N/A	0 (0.00)	0 (0.00)	N/A	0 (0.00)	15 (75.00)	N/A	0 (0.00)	0 (0.00)
	Resistant, *n* (%)	20 (100.00)	0 (0.00)	4 (20.00)	17 (85.00)	20 (100.00)	1 (5.00)	1 (5.00)	0 (0.00)	15 (75.00)
*S. haemolyticus* (6)	Susceptible, *n* (%)	4 (66.67)	6 (100.00)	6 (100.00)	5 (83.33)	6 (100.00)	6 (100.00)	5 (83.33)	6 (100.00)	2 (33.33)
	Intermediate, *n* (%)	N/A	0 (0.00)	0 (0.00)	N/A	0 (0.00)	0 (0.00)	N/A	0 (0.00)	0 (0.00)
	Resistant, *n* (%)	2 (33.33)	0 (0.00)	0 (0.00)	1 (16.67)	0 (0.00)	0 (0.00)	1 (16.67)	0 (0.00)	4 (66.67)
*S. pasteuri* (5)	Susceptible, *n* (%)	3 (60.00)	5 (100.00)	5 (100.00)	5 (100.00)	5 (100.00)	5 (100.00)	5 (100.00)	5 (100.00)	5 (100.00)
	Intermediate, *n* (%)	N/A	0 (0.00)	0 (0.00)	N/A	0 (0.00)	0 (0.00)	N/A	0 (0.00)	0 (0.00)
	Resistant, *n* (%)	2 (40.00)	0 (0.00)	0 (0.00)	0 (0.00)	0 (0.00)	0 (0.00)	0 (0.00)	0 (0.00)	0 (0.00)

Note: P 10: penicillin G 10 U, LNZ 30: linezolid 30 μg, SXT 25: trimethoprim 1.25 μg/sulphamethoxazole 23.75 μg, FA 10: fusidic acid 10 μg, NV 5: novobiocin 5 μg, DA 2: clindamycin 2 μg, FOX 30: cefoxitin 30 μg, RA 5: rifampicin 5 μg, and E 15: erythromycin 15 μg. N/A = not defined; no intermediate susceptibility category was defined according to CLSI ^a^ or EUCAST ^b^ criteria for the respective antibiotic. Novobiocin and penicillin G was excluded from MDR classification. Percentages were calculated using the number of tested isolates of each species as the denominator. The isolates were selectively sampled; these percentages do not estimate resistance prevalence among schoolchildren or among all recovered *Staphylococcus* isolates.

**Table 6:** Acquired antimicrobial resistance genes identified in *Staphylococcus arlettae* isolates using ResFinder.

Isolate	Resistance Gene	Antimicrobial Class	Coverage (%)	Identity (%)
208R	*mph(C)*	Erythromycin, Spiramycin, Telithromycin	100.00	100.00
	*dfrG*	Trimethoprim	100.00	100.00
	*msr(A)*	Erythromycin, Azithromycin, Telithromycin, Quinupristin, Pristinamycin ia, Virginiamycin s	100.00	98.98
	*msr(A)*	Erythromycin, Azithromycin, Telithromycin, Quinupristin, Pristinamycin ia, Virginiamycin s	100.00	86.78
209R(2)	*dfrG*	Trimethoprim	100.00	100.00
	*mph(C)*	Erythromycin, Spiramycin, Telithromycin	100.00	90.00
	*msr(A)*	Erythromycin, Azithromycin, Telithromycin, Quinupristin, Pristinamycin ia, Virginiamycin s	100.00	85.82%
508R(2)	*mph(C)*	Erythromycin, Spiramycin, Telithromycin	100.00	100.00
	*dfrG*	Trimethoprim	100.00	100.00
	*msr(A)*	Erythromycin, Azithromycin, Telithromycin, Quinupristin, Pristinamycin ia, Virginiamycin s	100.00	99.05%
	*msr(A)*	Erythromycin, Azithromycin, Telithromycin, Quinupristin, Pristinamycin ia, Virginiamycin s	100.00	86.78
603R(3)	*dfrG*	Trimethoprim	100.00	100.00
	*msr(A)*	Erythromycin, Azithromycin, Telithromycin, Quinupristin, Pristinamycin ia, Virginiamycin s	100.00	86.78

**Table 7:** Antimicrobial susceptibility phenotypes, acquired resistance genes, and genotype–phenotype concordance in selected *Staphylococcus arlettae* isolates.

Isolate	Resistant/Intermediate Antibiotics (AST)	Acquired Resistance Genes	Genotype–Phenotype Concordance
208R	Penicillin, trimethoprim/sulphamethoxazole, fusidic acid, clindamycin, erythromycin	*mph(C)*, *dfrG*, *msr(A)*	Partial concordance: the erythromycin-resistant phenotype was consistent with detection of *msr(A)* and *mph(C)*, and trimethoprim/sulfamethoxazole resistance was consistent with detection of *dfrG*, which confers resistance to the trimethoprim component. No corresponding acquired resistance determinants were detected by ResFinder for penicillin, fusidic acid or clindamycin.
209R(2)	Penicillin, trimethoprim/sulphamethoxazole, fusidic acid, clindamycin, erythromycin	*dfrG*, *mph(C)*, *msr(A)*	Partial concordance: the erythromycin-resistant phenotype was consistent with detection of *msr(A)* and *mph(C)*, and trimethoprim/sulfamethoxazole resistance was consistent with detection of *dfrG*, which confers resistance to the trimethoprim component. No corresponding acquired resistance determinants were detected by ResFinder for penicillin, fusidic acid or clindamycin.
508R(2)	Penicillin, trimethoprim/sulphamethoxazole, fusidic acid, clindamycin, erythromycin	*mph(C)*, *dfrG*, *msr(A)*	Partial concordance: the erythromycin-resistant phenotype was consistent with detection of *msr(A)* and *mph(C)*, and trimethoprim/sulfamethoxazole resistance was consistent with detection of *dfrG*, which confers resistance to the trimethoprim component. No corresponding acquired resistance determinants were detected by ResFinder for penicillin, fusidic acid or clindamycin.
603R(3)	Penicillin, trimethoprim/sulphamethoxazole, fusidic acid, clindamycin, erythromycin	*dfrG*, *msr(A)*	Partial concordance: the erythromycin-resistant phenotype was consistent with detection of *msr(A)*, and trimethoprim/sulfamethoxazole resistance was consistent with detection of *dfrG*, which confers resistance to the trimethoprim component. No corresponding acquired resistance determinants were detected by ResFinder for penicillin, fusidic acid or clindamycin.

Note: R, resistant; I, intermediate, according to the interpretive criteria described in Section 2.9. Absence of a ResFinder match indicates that no corresponding acquired resistance determinant was detected under the analysis conditions used; it does not establish the absence of a genetic resistance mechanism.

**Table 8:** Virulence-associated factors identified in *Staphylococcus arlettae* isolates using VirulenceFinder.

Isolate	Virulence-Associated Factor	Number of Hits	Identity (%)
208R	ACME	7	77.93–88.73
209R(2)	ACME	9	77.93–89.36
508R(2)	ACME	7	77.93–88.73
603R(3)	ACME	7	77.93–88.73

Note: The 137–634 bp matches were interpreted as partial ACME-associated sequences. These local sequence matches do not establish complete genes, an intact ACME, or its mobility or biological activity.

**Table 9:** Pathogenicity prediction of *Staphylococcus arlettae* isolates based on PathogenFinder2 analysis.

Isolate	Predicted Phenotype	Prediction Score (Mean ± SD)
208R	Human non-pathogenic	0.4357 ± 0.1131
209R(2)	Human non-pathogenic	0.3688 ± 0.1195
508R(2)	Human non-pathogenic	0.4538 ± 0.1176
603R(3)	Human non-pathogenic	0.3259 ± 0.1261

## Data Availability

The bacterial genome sequences have not been publicly deposited. The sequence files are maintained by the research team in the Department of Medical Microbiology and Parasitology, Universiti Sains Malaysia, under the supervision of Tuan Noorkorina Tuan Kub. The genomic data were generated from cultured bacterial isolates rather than participants’ human genomes. Enquiries concerning access may be directed to Tuan Noorkorina Tuan Kub; any proposed sharing will be considered within the applicable ethical approval and consent conditions.

## Supplementary Materials

The following supporting information can be downloaded at https://www.mdpi.com/article/10.3390/tropicalmed11100266/s1, File S1: Raw data for colony-forming unit counts, bacterial identification, and mould and yeast detection; File S2: Paired bacterial detection; File S3: Phenotypic antimicrobial susceptibility testing results; File S4: Archived outputs from the Center for Genomic Epidemiology bioinformatics analyses of four multidrug-resistant *Staphylococcus arlettae* isolates.

## Abbreviations

The following abbreviations are used in this manuscript:

ACME	Arginine catabolic mobile element
AMR	Antimicrobial resistance
AST	Antimicrobial susceptibility testing
BA	Blood agar
CDC	Centers for Disease Control and Prevention
CFUs	Colony-forming units
CGE	Center for Genomic Epidemiology
CLSI	Clinical and Laboratory Standards Institute
CoNS	Coagulase-negative *Staphylococcus* spp.
COVID-19	Coronavirus disease 2019
DCA	Deoxycholate Citrate Agar
EUCAST	European Committee on Antimicrobial Susceptibility Testing
MAC	MacConkey Agar
MALDI-TOF MS	Matrix-assisted laser desorption ionisation time-of-flight mass spectrometry
MDR	Multidrug-resistant
MRSA	Methicillin-resistant *Staphylococcus aureus*
MSA	Mannitol Salt Agar
MSSA	Methicillin-susceptible *Staphylococcus aureus*
P2 Solo	PromethION 2 Solo
PVL	Panton–Valentine leukocidin
Q1	First quartile
Q3	Third quartile
SD	Standard deviation
SIM	Sulphide–Indole–Motility
TNTC	Too numerous to count
TSI	Triple Sugar Iron
TSST-1	Toxic shock syndrome toxin-1
WGS	Whole-genome sequencing
WHO	World Health Organisation
