# Supplementary material for: Evaluation of Hand Bioburden and Genomic Features of Staphylococcus spp. Following Handwashing Practice Among Aboriginal Schoolchildren in Rural Malaysia
Source: Trop Med Infect Dis. 2026 Sep 25;11(10):266. doi: 10.3390/tropicalmed11100266 (PMC13709434; doi:10.3390/tropicalmed11100266)
Supplement: Supplementary file 1 [file tropicalmed-11-00266-s001.zip › File S2_Paired bacterial detection.pdf]

Exploratory paired comparisons of bacterial detection immediately before and after handwashing at the three-month follow-up visit among 60 Orang Asli schoolchildren.

Bacterial species	Before, n (%)	After, n (%)	Positive → negative, n	Negative → positive, n	Change, percentage points (95% CI)	Exact p-value
Gram-positive organism						
Staphylococcus aureus	7 (11.67)	1 (1.67)	6	0	-10.0 (-20.4, -1.4)	0.031
Staphylococcus saprophyticus	22 (36.67)	13 (21.67)	9	0	-15.0 (-24.6, -5.3)	0.004
Staphylooccus arlettae	18 (30.00)	12 (20.00)	6	0	-10.0 (-18.7, -1.6)	0.031
Staphylococcus ureilyticus	9 (15.00)	6 (10.00)	4	1	-5.0 (-14.3, 3.5)	0.375
Staphylococcus haemolyticus	6 (10.00)	1 (1.67)	5	0	-8.3 (-18.3, -0.2)	0.063
Staphylococcus pasteuri	5 (8.33)	5 (8.33)	3	3	0.0 (-9.8, 9.8)	1.000
Staphylococcus epidermidis	5 (8.33)	2 (3.33)	3	0	-5.0 (-13.9, 2.3)	0.250
Staphylococcus schweitzeri	1 (1.67)	0 (0.00)	1	0	-1.7 (-8.9, 4.5)	1.000
Staphylococcus sciuri	4 (6.67)	2 (3.33)	2	0	-3.3 (-11.6, 3.6)	0.500
Staphylococcus cohnii	2 (3.33)	1 (1.67)	1	0	-1.7 (-9.3, 5.1)	1.000
Staphylococcus kloosii	7 (11.67)	6 (10.00)	1	0	-1.7 (-8.4, 4.5)	1.000
Staphylococcus caprae	1 (1.67)	1 (1.67)	0	0	0.0 (-6.6, 6.6)	1.000
Staphylococcus lutrae	3 (5.00)	1 (1.67)	2	0	-3.3 (-1.8, 3.7)	0.500
Staphylococcus hominis	6 (10.00)	7 (11.67)	1	2	1.7 (-6.0, 9.7)	1.000
Staphylococcus nepalensis	5 (8.33)	3 (5.00)	2	0	-3.3 (-11.4, 3.5)	0.500
Staphylococcus capitis	1 (1.67)	1 (1.67)	0	0	0.0 (-6.6, 6.6)	1.000
Staphylococcus borealis	1 (1.67)	1 (1.67)	0	0	0.0 (-6.6, 6.6)	1.000
Staphylococcus muscae	1 (1.67)	1 (1.67)	0	0	0.0 (-6.6, 6.6)	1.000
Staphylococcus equorum	2 (3.33)	1 (1.67)	1	0	-1.7 (-9.3, 5.1)	1.000
Staphylococcus carnosus	2 (3.33)	2 (3.33)	0	0	0.0 (-6.4, 6.4)	1.000

Staphylococcus warneri	2 (3.33)	4 (6.67)	1	3	3.3 (-5.2, 12.6)	0.625
Staphylococcus xylosus	3 (5.00)	2 (3.33)	1	0	-1.7 (-9.2, 5.0)	1.000
Staphylococcus sp.	0 (0.00)	4 (6.67)	0	4	-1.7 (-0.6, 15.9)	0.125
Gram-negative organism						
E.coli/ Shigella complex	21 (35.0)	0 (0.00)	21	0	-35.0 (-47.6, -22.6)	<0.001
Klebsiella pneumoniae	6 (10.00)	0 (0.00)	6	0	-10.0 (-20.1, -2.0)	0.031
Acinetobacter variabilis	8 (13.33)	5 (8.33)	7	4	-5.0 (-16.6, 6.3)	0.549
Acinetobacter indicus	2 (3.33)	1 (1.67)	2	1	-1.7 (-9.8, 6.0)	1.000
Acinetobacter baumannii	6 (10.00)	1 (1.67)	6	1	-8.3 (-18.6, 0.8)	0.125
Acinetobacter ursingii	1 (1.67)	0 (0.00)	1	0	-1.7 (-8.9, 4.5)	1.000
Acinetobacter schindleri	1 (1.67)	4 (6.67)	1	4	5.0 (-3.4, 14.4)	0.375
Acinetobacter nosocomialis	1 (1.67)	1 (1.67)	1	1	0.0 (-7.3, 7.3)	1.000
Acinetobacter radioresistens	1 (1.67)	0 (0.00)	1	0	-1.7 (-8.9, 4.5)	1.000
Acinetobacter sp.	1 (1.67)	0 (0.00)	1	0	-1.7 (-8.9, 4.5)	1.000
Enterobacter cloacae	2 (3.33)	0 (0.00)	2	0	-3.3 (-11.4, 3.2)	0.500
Moraxella osloensis	11 (18.33)	16 (26.67)	8	13	8.3 (-6.6, 22.9)	0.383
Massilia varians	1 (1.67)	2 (3.33)	0	1	1.7 (-5.1, 9.3)	1.000
Massilia timonae	0 (0.00)	1 (1.67)	0	1	1.7 (-4.5, 8.9)	1.000
Stenotrophomonas maltophilia	2 (3.33)	0 (0.00)	2	0	-3.3 (-11.4, 3.2)	0.500
Pseudomonas aeruginosa	1 (1.67)	0 (0.00)	1	0	-1.7 (-8.9, 4.5)	1.000
Pseudomonas oryzihabitans	1 (1.67)	0 (0.00)	1	0	-1.7 (-8.9, 4.5)	1.000
Pseudomonas stutzeri	0 (0.00)	1 (1.67)	0	1	1.7 (-4.5, 8.9)	1.000
Pseudomonas sp.	0 (0.00)	1 (1.67)	0	1	1.7 (-4.5, 8.9)	1.000
Brevundimonas diminuta	0 (0.00)	1 (1.67)	0	1	1.7 (-4.5, 8.9)	1.000
Yersinia enterocolitica	1 (1.67)	0 (0.00)	1	0	-1.7 (-8.9, 4.5)	1.000

Note: Data are presented as numbers and percentages of participants, with 60 participants providing paired observations. Positive → negative denotes participants in whom an organism was detected before but not after handwashing; negative → positive denotes participants in whom it was detected after but not before handwashing. Change in detection proportion was calculated as after minus before and expressed in percentage points: [(negative → positive) − (positive → negative)]/60 × 100. Negative values indicate decreased detection and positive values indicate increased detection.

Species-level comparisons were exploratory and used two-sided exact McNemar tests based on discordant pairs. The p-values shown are unadjusted; where there were no discordant pairs, p = 1.000 is reported. Confidence intervals are unadjusted 95% estimation intervals. Statistical significance was assessed using the Holm-adjusted p-values reported in Table 4, with adjustment across the 52 microbiological comparisons described in the Methods. Detection denotes recovery under the study's culture and identification procedures and does not establish persistent carriage, colonisation or transmission. CI, confidence interval.
